# The Rehbinder Effect in Testing Saturated Carbonate Geomaterials

**DOI:** 10.3390/ma16083024

**Published:** 2023-04-11

**Authors:** Evgenii Riabokon, Mikhail Turbakov, Evgenii Kozhevnikov, Vladimir Poplygin, Hongwen Jing

**Affiliations:** 1 Department of Oil and Gas Technologies, Perm National Research Polytechnic University, 614990 Perm, Russia; msturbakov@gmail.com (M.T.); kozhevnikov_evg@mail.ru (E.K.);; 2 State Key Laboratory for Geomechanics and Deep Underground Engineering, China University of Mining and Technology, Xuzhou 221116, China

**Keywords:** geomaterial, carbonate rock, strength, saturation, Rehbinder effect

## Abstract

Carbonate geomaterial samples were tested for uniaxial compressive strength and tensile strength under air-dried and distilled-water-wet conditions. When tested for uniaxial compression, samples saturated with distilled water showed 20% lower average strength than that of air-dried samples. In the indirect tensile (Brazilian) test, samples saturated with distilled water showed 25% lower average strength than that of dry samples. In comparison with air-dried conditions, when the geomaterial is saturated with water, the ratio of the tensile strength to the compressive strength is decreased, mainly due to the decrease in the tensile strength caused by the Rehbinder effect.

## 1. Introduction

Depending on the saturating medium and the degree of saturation, the strength of a geomaterial differs. The uniaxial compressive strength, the uniaxial tensile strength and the Brazilian tensile strength are lower in wet conditions than in dry ones [1]. There are several mechanisms that describe rock’s strength reduction with moisture [2]. It was revealed [3] in tests of Sanjome andesite that its uniaxial compressive strength decreases as water saturation increases and the loading rate increases. Based on a large number of experiments using sandstone samples, it was found [4] that the moisture content strongly affects the strength of the rock under uniaxial compression, which is mostly related to pore radius distribution, poor matrix mineralogy and the amount of cement. It was proved [5] that the principal mechanism causing the water weakening of the chalk is related to the added pressure on the grains caused by the attraction of the water molecules to the chalk’s surface. It is also known—for example, from [6]—that during drying, rocks (e.g., sandstones) saturated with water recover their mechanical properties and strength as in the dry state, and that when saturated with water, pore pressure plays a limited role in affecting the mechanical properties of sandstones. It was demonstrated experimentally [1] that the tensile strengths of Sanjome andesite, Tage tuf and Kimachi sandstone determined in Brazilian tests decrease with water saturation. A review of experimental works in terms of rock types [7] showed that the largest portion of researchers’ attention is paid to sedimentary rocks and sandstones in particular. Modern studies have demonstrated the ability of the discrete element method to model heterogeneous geomaterials and their mechanical characteristics [8,9], and the ability of the lattice Boltzmann method to model free-surface granular flow [10].

Among the studies of carbonate sedimentary rocks, it is worth noting the early work [11], which shows that the saturation of Solnhofen limestone with water at room temperature leads to a decrease in its strength. Using Indiana limestone, the authors of [12] studied the effects of saturation with water, glycerine, ethylene glycol, nitrobenzene, ethyl alcohol, benzaldehyde and n-butyl alcohol on its strength characteristics and postulated that the effect of a fluid saturating the rock is to change the surface free energy of the rock and hence its strength. The greater the surface tension of the saturating fluid and the dielectric constant, the lower the adhesion (and hence the strength) between the particles that make up the geomaterial. In more recent work [13], the authors obtained significant decreases in the uniaxial compressive strengths and elastic moduli of water-saturated limestone samples compared to dry ones, justifying the theory that thermal radiation depends on the degree of water saturation of rocks. The results of another study [14] suggested that water weakens the bonding strength of any rock’s structure. A suction approach was developed [15] to reduce the strengths of Carboniferous and Magnesian limestones through an increase in saturation with moisture, associated with the high surface tension of water. A study on calcareous porous rocks [16] postulated that its long- and short-term hydrochemomechanical weakening are caused by the formation of two distinct types of bonding within the rock. At the same time, it was pointed out [6] that there are several combined factors (mechanisms) that govern the rock deformation, and some factors are more significant than others for certain rock types and conditions. At the same time, the major mineral phase in carbonate rocks, and limestones in particular, is calcite, which is a crystalline material.

An experimental study [17] revealed that the strength of a rock is affected by its polycrystalline microstructure. It was found [18] that liquids reducing the free surface energy of rocks can cause a noticeable change in their mechanical properties. Such a change is known as the Rehbinder effect, which is described in [19,20]. The Rehbinder effect is useful in the drilling [21] and cutting [22] of rocks. It was found [23] that a decrease in the surface energy of calcium is caused by the polar interaction between grain surfaces and water. Later, a molecular dynamic study [24] showed that wetting reduces the surface energy of calcite and leads to calcite surface rearrangement, which finally affects the strength of limestone.

Following the Rehbinder theory, it is assumed that for crystalline geomaterials (such as limestones), the effect of a decrease in the tensile strength should be more noticeable than in other sedimentary reservoir rocks. Thus, the values of the ratio of the uniaxial compressive strength to the tensile strength in different saturation conditions for limestones need not be the same (not sensitive to a degree of saturation), as stated in [25] for Hungarian Miocene sandstone samples.

Despite a large number of works devoted to the study of the effects of the saturation of sedimentary rocks with water on their strength under various conditions, the question of the effect of saturation on the ratio of the tensile strength to the uniaxial compressive strength of carbonate geomaterials remains not fully answered.

In this regard, the purpose of this work was to study the effect of moisture on the ratio of the tensile strength to the uniaxial compressive strength of such a carbonate geomaterial as a limestone.

## 2. Materials and Methods

### 2.1. Description of Materials

**Figure 1:**
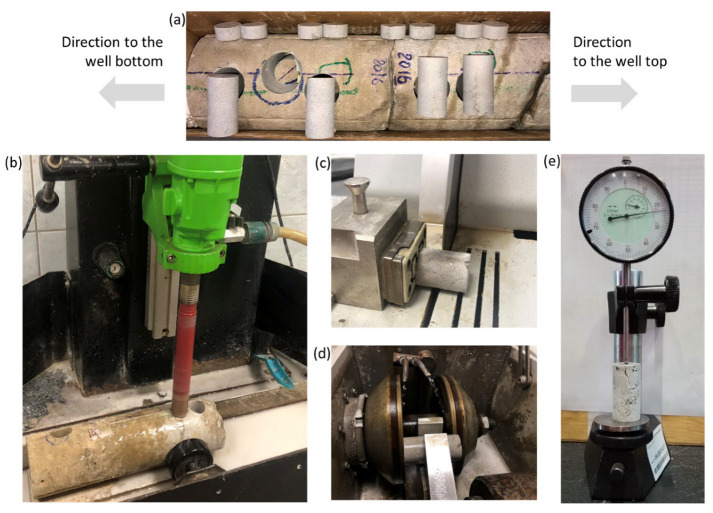
Photographs depicting the sample preparation steps: (**a**) the core from a production well; (**b**) drilling out the sample; (**c**) cutting the sample; (**d**) grinding the ends of the sample; (**e**) sample measurement.

The initial material for samples was a core that was 100 mm in diameter, sourced from 2 km deep in a production well located in an oil field in Perm region (Figure 1a). The geomaterial belongs to Bashkirian layer of Middle Carboniferous sediments.

During manufacture, the samples went through the common stages of preparation of rock samples for mechanical testing, such as drilling, cutting and grinding. In the first stage, samples were drilled from the core parallel to the rock. The sample axis was orthogonal to the well axis. Drilling was carried out with diamond bits with a diameter of 25.4 mm (Figure 1b). Flushing was carried out at all stages of sample preparation to cool the rock and remove cuttings. In the second stage, the drilled fragments were cut out on a disk machine (Figure 1c). The surfaces at the ends of the specimens were ground and polished so that they were mirror-like (Figure 1d). The dimensions of specimens then were measured (see Figure 1e), and specimens were either accepted for further study or burnt-out if they did to meet requirements of the standards ASTM D4543-19 [26] and ISRM 0020-7624 [27]. Prepared samples also went through the extraction of hydrocarbons with help of a Soxhlet apparatus.

**Figure 2:**
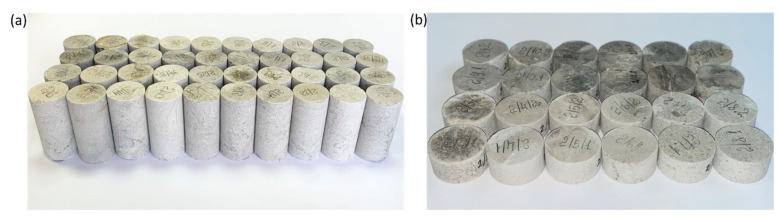
Photographs depicting prepared limestone samples: (**a**) samples 50.8 mm in height and 25.4 mm in diameter; (**b**) samples 12.7 mm in thickness and 25.4 mm in diameter.

In total, 42 samples of a geomaterial 50.8 mm long and 25.4 mm in diameter and 24 samples 12.7 mm thick and 25.4 mm in diameter were prepared (Figure 2). The limestone is dense: porosity of 2% and permeability of 0.015 μm^2^. In accordance with Recommended Practice 40 of the American Petroleum Institute [28], samples were washed and dried in an oven; as a result, an air-dried state was achieved.

**Figure 3:**
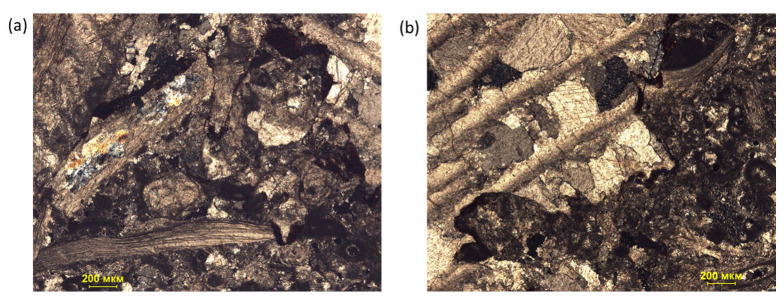
Microphotographs of thin sections of two samples: (**a**) fragment sectioned with an analyzer depicting brachiopod shells; (**b**) fragment of a bryozoan, the chamber of which is filled with medium-grained calcite.

According to the analysis of a thin section, the components of the limestone were represented by bioclasts composed of micritic calcite and clear-crystalline calcite (see Figure 3). The chambers of the micritic matrix were filled with quartz. The secondary calcite cement comprised different crystals with grains ranging from 0.08 to 0.3 mm in size. The pores were formed between crystals due to the process of recrystallization.

### 2.2. Description of Methods

**Figure 4:**
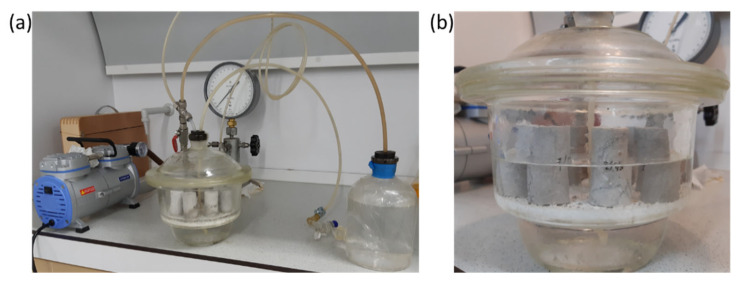
Photographs depicting the process of saturation of geomaterial samples: (**a**) sample saturation unit AST-600; (**b**) limestone samples during vacuum saturation.

To study the effects of distilled water saturation on the uniaxial compressive strength and the tensile strength—knowing that the reservoir properties of the geomaterial under study are poor, and in order to create full saturation of samples, including their cores—the process of saturation was performed over 24 h under vacuum conditions using an automatic saturation unit, AST-600 (Figure 4), which allowed full imbibition of the rock, including its fine pores.

**Figure 5:**
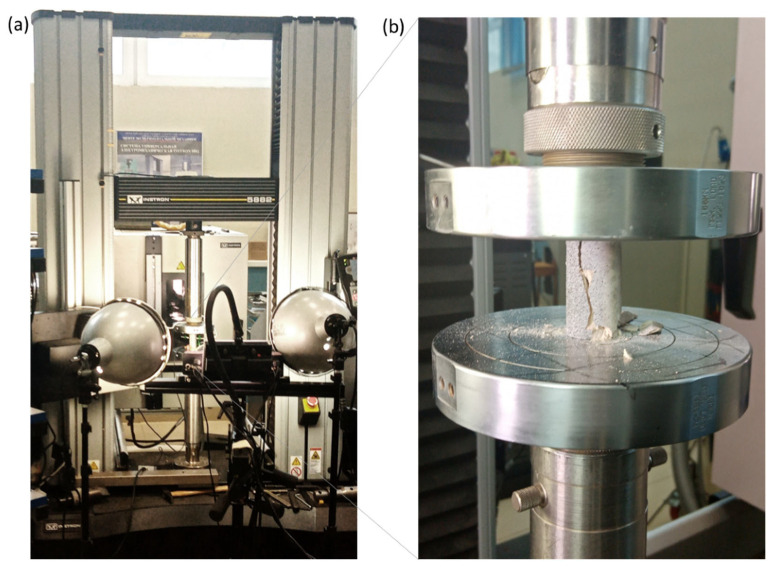
Photographs depicting the uniaxial compression test of the geomaterial: (**a**) Instron 5882 universal electromechanical system with the installed Vic 3D strain field registration system; (**b**) the tested sample of geomaterial between the loading plates at the final stage of loading-brittle fracture in the shear mode along a single plane.

Testing of samples was performed on an Instron 5882 universal electromechanical system (Figure 5). The longitudinal (axial) strain of the samples was recorded using the Vic 3D strain field registration system, which included two video cameras with high resolution and recording frequency. The rate of movement of the loading plate in the tests was 0.1 mm/min.

## 3. Results and Discussion

### 3.1. Testing Dry and Saturated Samples for Uniaxial Compressive Strength

**Figure 6:**
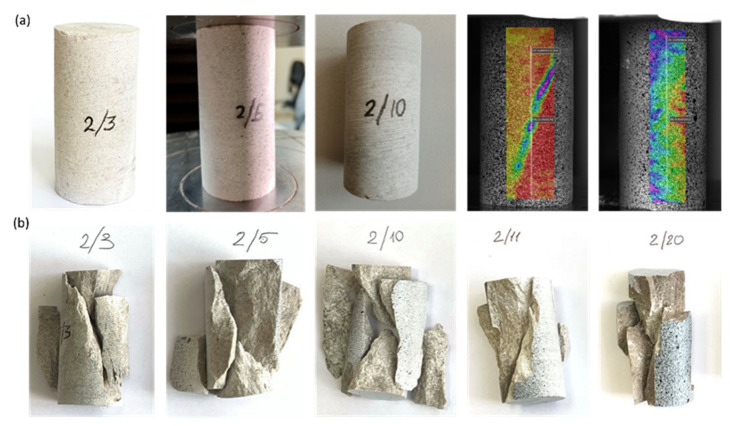
Photographs depicting air-dried samples during testing for uniaxial compression: (**a**) samples before failure; (**b**) samples after testing demonstrating different failure modes, such as Y-shaped (samples 2/3, 2/5 and 2/20), double shear (sample 2/10) and shear along a single plane (sample 2/11).

In uniaxial compression tests, the strength of air-dried samples and the maximum load were determined. Before testing, the samples were photographed (see samples 2/3, 2/10 in Figure 6a) and installed between the plates on the test system (see sample 2/5), and the samples were also endowed with a speckle structure (painted), which with the help of a Vic 3D strain field registration system, allowed us to track the deformation of samples under loading (see samples 2/11 and 2/20). For example, the observed displacement of the regions of the speckle structure applied to sample 2/11 allowed identification of signs of future shear along one plane, as shown in Figure 6b, which also demonstrates the conventional failure modes of other samples obtained as a result of testing.

**Figure 7:**
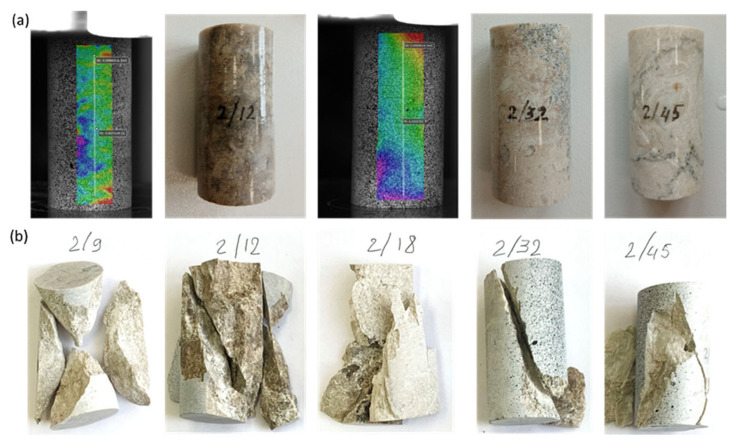
Photographs depicting water-wet samples during testing for uniaxial compression: (**a**) samples before failure; (**b**) samples after testing demonstrating different failure modes, such as double shear (sample 2/9), shear along a single plane (samples 2/12, 2/32, 2/45) and Y-shaped (sample 2/18).

Similarly, samples saturated with distilled water were studied for uniaxial compression (Figure 7a). Samples 2/18, 2/32 and 2/45 were painted with speckles before testing. During the loading test, the standard failure modes of the samples were also observed, as shown in Figure 7b.

The testing machine provided the results in coordinates F−Δl (load–displacement). Thus, in order to get loading diagrams in coordinates σ−ε (stress–strain), the data were recalculated using the formulas: σ=F/S, where S refers to a sample cross-sectional area, and ε=Δl/l, in accordance with the standard ASTM D7012-04 [29].

**Figure 8:**
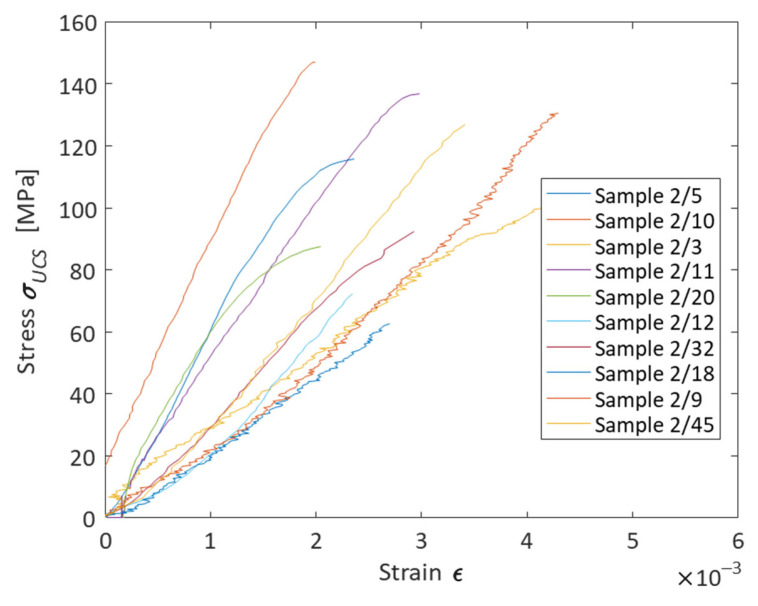
A loading diagram for ten cylindrical specimens of a geomaterial saturated with air or distilled water showing stress–strain coordinates.

The result of the recalculation was the building of a loading diagram for each sample (Figure 8) (Table 1).

As a result of testing the samples for uniaxial compression, it was revealed that samples saturated with distilled water are characterized by less strength than samples saturated with air. Diagram 8 shows that the strength values of rock saturated with air ranged from 87.6 to 147.1 MPa. The average as 118.7 MPa. The strength of samples saturated with water ranged from 62.7 to 130.6 MPa. The average value was 97.0 MPa. This indicates that when the phase saturating the geomaterial is changed from air to distilled water, the strength of the rock decreases two-fold.

### 3.2. Testing Dry and Saturated Samples for the Tensile Strength

**Figure 9:**
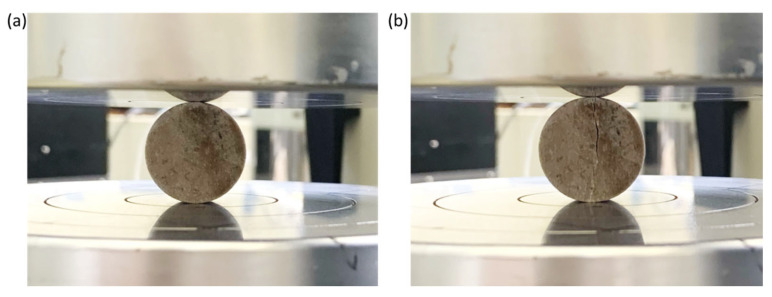
Photographs depicting rock sample 2/5/2 between loading plates: (**a**) before loading; (**b**) after loading.

The testing of disk samples was carried out according to the indirect method (Brazilian test), in which a rock sample was subjected to diametrical loading along a cylindrical plane at a speed of 0.1 mm/min. The classic failure mode of samples in the Brazilian test is the formation of a longitudinal crack (Figure 9).

**Figure 10:**
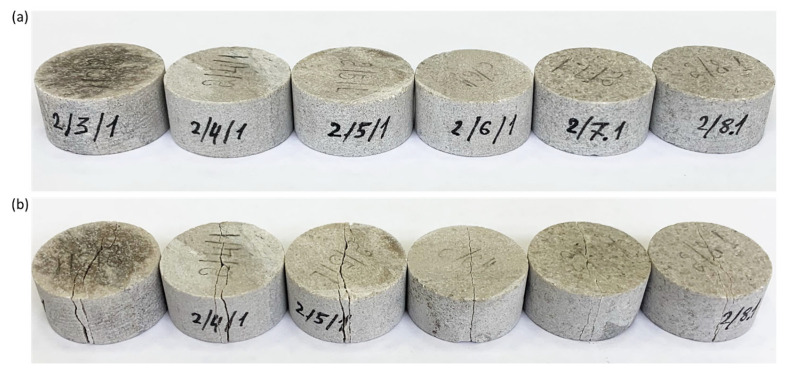
Photographs depicting air-dried limestone samples during indirect determination of the tensile strength throughout the Brazilian test: (**a**) samples before testing; (**b**) samples after testing.

**Figure 11:**
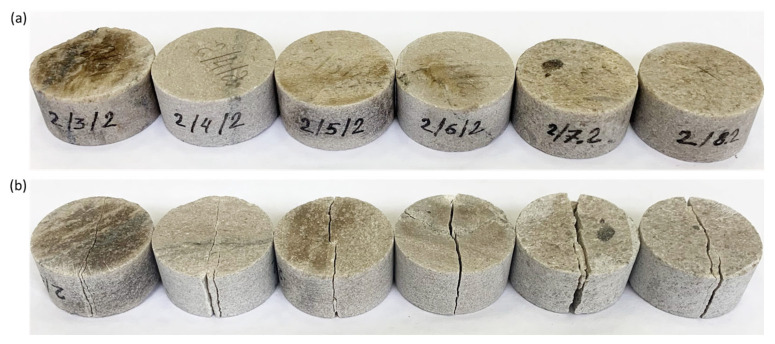
Photographs depicting water-wet limestone samples during indirect determination of the tensile strength through the Brazilian test: (**a**) samples before testing; (**b**) samples after testing.

**Figure 12:**
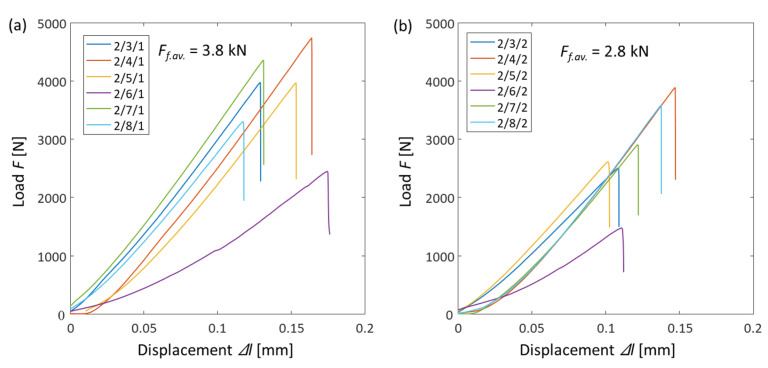
Diagrams of loading the geomaterial disk samples with indirect determination of the tensile strength: (**a**) air-dried samples; (**b**) water-wet samples.

As a result of testing in samples in the direction parallel to the application of the load, cracks appeared between the points at which there was contact between the disk and the loading plates. In all air-dry and water-wet samples, a similar deformation mode was observed in the form of a central crack (see Figure 10 and Figure 11). The loading of sample 2/6/2 featured the activation of a stylolite weld (see Figure 11b), as a result of which the load at failure for the sample was lower than those of other samples (see Figure 12).

Note that despite the existence of many different failure modes [30], in the present study, the similar failure modes were most likely due to the absence of bedding layers in the limestone and the high density and strength of the rock. At the same time, the loading diagrams (Figure 12) show that the nature of the loading of the samples was not the same. This was due to the heterogeneity of the geomaterial and the presence in the rock of inclusions of various types and sizes, which were clearly visible with microscopy (Figure 3). As a result, samples taken from the one geomaterial core showed some scattering in tensile-strength values.

Knowing the load valued at the moment of fracture $F_{f}$, diameter D and thickness t of the sample, the tensile strength $\sigma_{BTS}$ can be calculated using the formula $\sigma_{BTS}=0.636\times F_{f}/Dt$ in accordance with ISRM 0020-7624 (Table 2).

Test results show that when this limestone is saturated with distilled water, the ratio of the tensile strength to compressive strength increases. This phenomenon can be associated with the Rehbinder effect, according to which, in presence of tensile stresses and having the water as a saturating phase, the strength of the geomaterial $\sigma_{BTS\;wet.}$ decreases more significantly than in tensile tests with air-dried samples: $\frac{\sigma_{BTS\;dry}}{\sigma_{UCS\;dry}}\to \frac{\sigma_{BTS\;wet}}{\sigma_{UCS\;wet}}$. When substituting in the values, we obtain $\frac{7.43}{118.7}=0.063\to \frac{5.53}{97.0}=0.057,$ which corresponds to a reduction in the ratio of the tensile strength to compressive strength during the transition from air-dried to water-wet conditions by 9%.

**Figure 13:**
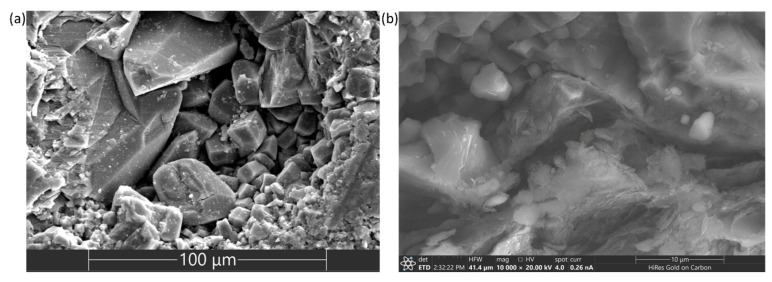
Electron microphotographs showing the structure of carbonate geomaterial: (**a**) various calcite crystal shapes, such as rhombohedral, scalenohedral (the large crystal at top center) and crystals of other types; (**b**) a system of microcracks.

According to the Rehbinder effect, as a result of adsorption of water in rock pores and the fracture surface, changes in the mechanical properties of a geomaterial occurs due to physicochemical processes that cause a decrease in the surface (interfacial) energy of the rock. Developing the theory of the Rehbinder effect, it can be assumed that due to the complex polycrystalline structure of minerals comprising the limestones in the presence of water, cracks between crystals (grains) can propagate throughout the fiber of the geomaterial. Considering that the structure of the limestone consists of sets of cracks developed at different scales (from microscale to mesoscale), their propagation lowers the strength of a rock material in the presence of acting forces. Microphotographs obtained by electron microscopy indicate the presence of cracks and crystalline systems in limestones at different levels (Figure 13) and the areas of recrystallization.

Therefore, when a carbonate geomaterial is fully saturated with water, an additional contribution associated with the formation of new disconnections is made to the decrease in rock strength caused by a decrease in surface energy. This is also supported by the results of some studies [31], according to which, the level of saturation of the limestone sample determines the character of the distribution of macrocracks on the inside of the sample.

Among the typical characteristics for the Rehbinder effect, strength reduction, embrittlement and enhancement of solid plasticity can be highlighted, as they facilitate rock disintegration and grinding [32]. Typical requirements for the Rehbinder effect to be manifested are the material being of a crystalline nature, the presence of a wetting phase (e.g., water) coating the rock and the presence of tensile stresses acting inside the rock (due to e.g., applied force) [20]. As was argued by the Rehbinder himself and by Shchukin [33], and by the authors in further studies [34], drops of water coat rock grains and crystals and reduce their surface energy. If rock is subjected to stress, such a reduction in surface energy (strength of bonds among grains, crystal and cementing matter) will make a significant contribution to the decrease in strength and make the rock more pliable.

Another reason why the rock’s strength weakens in the presence of a wetting phase is due to chemomechanical effects (e.g., dissolution) [35]. However, chemomechanical effects are usually observed between reactive materials. In our study, the rock extracted from the subsoil was initially saturated with water. Thus, it was not expected that the rock would exert, in laboratory conditions, any behavior that is atypical for reservoir conditions. Even if chemomechanical effects manifest, they would not likely contribute to the decrease in the rock’s strength more than the Rehbinder effect.

The fact that the ratio of the tensile strength _𝜎𝐵𝑇𝑆 𝑤𝑒𝑡_ to the uniaxial compressive strength 𝜎_𝑈𝐶𝑆 𝑤𝑒𝑡_ of the water-wet samples is much lower than the ratio of the tensile strength 𝜎_𝐵𝑇𝑆 𝑑𝑟𝑦_ to the uniaxial compressive strength 𝜎_𝑈𝐶𝑆 𝑑𝑟𝑦_ of the air-dried samples, crystals of various shapes (comprising the limestone’s structure) and microcracks are the criteria which support the Rehbinder theory.

Taking into account that a small amount of moisture is sufficient for the manifestation of the Rehbinder effect, in previous studies, in experiments on the dynamic loading of limestone [36], in which geomaterial samples were completely saturated, we were able to assume that the values of the Young’s modulus shrank as frequency increased, and were low compared with the situation of similar dynamic loading of dry samples (a decrease in the Young’s modulus as the water saturation increased was also reported). Such an effect was also observed in other similar studies [37,38,39].

## 4. Conclusions

In this work, an experimental study of the effects of the saturation of a carbonate rock with distilled water and air on the strength of samples under uniaxial compression and indirect tension was carried out. We used a dense limestone extracted from a production well. The microstructure of the carbonate geomaterial was studied before the mechanical tests. Based on the results of testing, the following conclusions can be drawn:

(1) The uniaxial compressive strength and the indirect tensile strength of Bashkir limestone are significantly reduced when the sample is fully saturated with distilled water. In comparison with dry samples, when saturated with water, the uniaxial compressive strength decreased from 118.7 to 97.0 MPa, and the tensile strength decreased from 7.43 to 5.53 MPa.
(2) The ratio of the indirect tensile strength to the uniaxial compressive strength decreased by 9% at full saturation of samples with distilled water compared to air-dried samples, which is associated with the Rehbinder effect, which is especially strong in a polycrystalline rock with microcracks such as limestone, and in the presence of tensile stresses.
(3) The research results confirmed the results of previous studies, according to which the strength of carbonate rocks can significantly decrease when they are saturated with water, and also expanded them in terms of explaining the reason for this decrease.

## Figures and Tables

**Table 1:** Results of the uniaxial compression tests of limestone geomaterial samples. Data on air-dried samples are in the top rows, and data on water-wet samples are in the bottom rows.

Sample	Diameter D, mm	Length l, mm	$\mathrm{Load}\;\mathrm{at}\;\mathrm{Failure}\;F_{f}$, kN	$\sigma_{UCS}$, MPa	$\sigma_{UCS\;av.}$, MPa
2/3	25.4	50.8	53.8	106.2	118.7
2/5	25.4	50.8	58.6	115.7	
2/10	25.4	50.8	74.5	147.1	
2/11	25.4	50.8	69.3	136.9	
2/20	25.4	50.8	44.3	87.6	
2/9	25.4	50.8	66.2	130.6	97.0
2/12	25.4	50.8	36.6	72.3	
2/18	25.4	50.8	31.7	62.7	
2/32	25.4	50.8	46.8	92.4	
2/45	25.4	50.8	64.3	126.9	

**Table 2:** Results of the indirect tensile tests of limestone geomaterial samples. Data on air-dried samples are in the top rows, and data on water-wet samples are in the bottom rows.

Sample	Diameter D, mm	Thickness t, mm	$\mathrm{Load}\;\mathrm{at}\;\mathrm{Failure}\;F_{f}$, kN	$\sigma_{BTS}$, MPa	$\sigma_{BTS\;av.}$, MPa
2/3/1	25.4	12.7	3.9	7.8	7.4
2/4/1	25.4	12.7	4.7	9.3	
2/5/1	25.4	12.7	3.9	7.8	
2/6/1	25.4	12.7	2.4	4.8	
2/7/1	25.4	12.7	4.3	8.5	
2/8/1	25.4	12.7	3.2	6.4	
2/3/2	25.4	12.7	2.5	4.9	5.5
2/4/2	25.4	12.7	3.8	7.6	
2/5/2	25.4	12.7	2.6	5.1	
2/6/2	25.4	12.7	1.4	2.9	
2/7/2	25.4	12.7	2.9	5.7	
2/8/2	25.4	12.7	3.5	7.0	

## Data Availability

Not applicable.

## References

[1] Bao T., Hashiba K., Fukui K. (2021). Effect of Water Saturation on the Brazilian Tension Test of Rocks. Mater. Trans..

[2] Van Eeckhout E.M. (1976). The mechanisms of strength reduction due to moisture in coal mine shales. Int. J. Rock Mech. Min. Sci. Geomech. Abstr..

[3] Hashiba K., Fukui K., Kataoka M. (2019). Effects of water saturation on the strength and loading-rate dependence of andesite. Int. J. Rock Mech. Min. Sci..

[4] Demarco M.M., Jahns E., Rüdrich J., Oyhantcabal P., Siegesmund S. (2007). The impact of partial water saturation on rock strength: An experimental study on sandstone. Z. Dtsch. Ges. Geowiss..

[5] Risnes R., Madland M.V., Hole M., Kwabiah N.K. (2005). Water weakening of chalk—Mechanical effects of water–glycol mixtures. J. Pet. Sci. Eng..

[6] Zhou Z., Cai X., Cao WLi X., Xiong C. (2016). Influence of Water Content on Mechanical Properties of Rock in Both Saturation and Drying Processes. Rock Mech. Rock Eng..

[7] Yasar S. (2020). Long term wetting characteristics and saturation induced strength reduction of some igneous rocks. Environ. Earth Sci..

[8] Lin Y.X., Lai Z.S., Ma J.J., Huang L.C., Lei M.F. (2023). A FDEM approach to study mechanical and fracturing responses of geo-materials with high inclusion contents using a novel reconstruction strategy. Eng. Fract. Mech..

[9] Huang S., Huang L.C., Lai Z.S., Zhao J.D. (2023). Morphology characterization and discrete element modeling of coral sand with intraparticle voids. Eng. Geol..

[10] Yang G.C., Yang L., Kwok C.Y., Sobrai Y.D. (2023). Efficient lattice Boltzmann simulation of free-surface granular flows with μ(I)-rheology. J. Comput. Phys..

[11] Rutter E.H. (1974). The influence of temperature, strain rate and interstitial water in the experimental deformation of calcite rocks. Tectonophysics.

[12] Vutukuri V.S. (1974). The effect of liquids on the tensile strength of limestone. Int. J. Rock Mech. Min. Sci. Geomech. Abstr..

[13] Blokhin D.I., Ivanov P.N., Dudchenko O.L. (2021). Experimental study of thermomechanical effects in water-saturated limestones during their deformation. J. Min. Inst..

[14] Michalopoulos L.P., Triandafilidis G.E. (1976). Influence of Water on Hardness, Strength and Compressibility of Rock. Environ. Eng. Geosci..

[15] West G. (1994). Effect of suction on the strength of rock. Q. J. Eng. Geol. Hydrogeol..

[16] Ciantia M.O., Castellanza R., Crosta G.B., Hueckel T. (2015). Effects of mineral suspension and dissolution on strength and compressibility of soft carbonate rocks. Eng. Geol..

[17] Ji X., Zhou W., Chen Y., Ma G. Generation of the Polycrystalline Rock Microstructure by a Novel Voronoi Grain-Based Model With Particle Growth. Proceedings of the 52nd U.S. Rock Mechanics/Geomechanics Symposium.

[18] Traskin V.Y. (2009). Rehbinder effect in tectonophysics. Izv. Phys. Solid Earth.

[19] Rehbinder P.A., Lichtman V. (1957). Effect of surface active media on strain and rupture in solids. Proc. Second. Int. Congr. Surf. Act..

[20] Rehbinder P.A. (1979). On the Influence of Changes in the Surface Energy on the Cleavage, Hardness and Other Properties of Crystals, in P. A. Rehbinder. Selected Transactions. Physicochem. Mech..

[21] Rehbinder P.A., Shreiner L.A., Zhigach K.F. (1979). The Hardness Reducers in Drilling,” in P.A. Rehbinder. Selected Transactions. Physicochem. Mech..

[22] Yadav S., Hagan P., Naj A., Bob K. The effect of water saturation in sandstone and limestone samples on disc cutting performances. Proceedings of the 17th Coal Operators’ Conference.

[23] Boozer G.D., Hiller K.H., Serdengecti S. Effects of pore fluids on the deformation behavior of rocks subjected to triaxial compression. Proceedings of the 5th Symposium on Rock Mechanics, University of Minnesota.

[24] Kvamme B., Kuznetsova T., Uppstad D. (2009). Modelling excess surface energy in dry and wetted calcite systems. J. Math. Chem..

[25] Vásárhelyi B., Davarpanah M. (2018). Influence of Water Content on the Mechanical Parameters of the Intact Rock and Rock Mass. Period. Polytech. Civ. Eng..

[26] (2019). Standard Practices for Preparing Rock Core as Cylindrical Test Specimens and Verifying Conformance to Dimensional and Shape Tolerances.

[27] Bieniawski Z.T., Hawkes I. (1978). ISRM Suggested Methods for Determining Tensile Strength of Rock Materials Part 2: Suggested Method for determining indirect tensile strength by the Brazil Test. Int. J. Rock Mech. Min. Sci. Geomech. Abstr..

[28] American Petroleum Institute (1998). Recommended Practices for Core Analysis. Recommended Practice 40.

[29] (2017). Standard Test Method for Compressive Strength and Elastic Moduli of Intact Rock Core Specimens under Varying States of Stress and Temperatures.

[30] Basu A., Mishra D.A., Roychowdhury K. (2013). Rock failure modes under uniaxial compression, Brazilian, and point load tests. Bull. Eng. Geol. Environ..

[31] Wen W., Shiwei Z., Shen W., Dongyin L., Wei W., Yuxi H., Heng W. (2021). Mechanical behavior of limestone in natural and forced saturation states under uniaxial loading: An experimental study. Geomech. Geophys. Geo-Energy Geo-Resour..

[32] Sokur N., Biletskyy VSokur L., Bozyk D., Sokur I. (2016). Investigation of the process of crushing solid materials in the centrifugal disintegrators. East. -Eur. J. Enterp. Technol..

[33] Rehbinder P.A., Shchukin E.D. (1972). Surface phenomena in solids during deformation and fracture processes. Prog. Surf. Sci..

[34] Bergsaker A.S., Røyne A., Ougier-Simonin A., Aubry J., Renard F. (2016). The effect offluid composition, salinity, and acidity on subcriticalcrack growth in calcite crystals. J. Geophys. Res. Solid Earth.

[35] Voigtländer A., Leith K., Krautblatter M. (2018). Subcritical crack growth andprogressive failure in Carrara marbleunder wet and dry conditions. J. Geophys. Res. Solid Earth.

[36] Riabokon E., Turbakov M., Popov N., Kozhevnikov E., Poplygin V., Guzev M. (2021). Study of the Influence of Nonlinear Dynamic Loads on Elastic Modulus of Carbonate Reservoir Rocks. Energies.

[37] Guzev M.A., Kozhevnikov E.V., Turbakov M.S., Riabokon E.P., Poplygin V.V. (2021). Experimental investigation of the change of elastic moduli of clastic rocks under nonlinear loading. Int. J. Eng. Trans. C Asp..

[38] Guzev M.A., Riabokon E.P., Turbakov M.S., Kozhevnikov E.V., Poplygin V.V. Study on the effect of nonlinear dynamic loads on the elastic modulus of rocks during hydrocarbon fields development. Proceedings of the 7th Scientific Exploration Conference—Tyumen 2021: Natural Resources Management as a Cross-Functional Process.

[39] Riabokon E., Poplygin V., Turbakov M., Kozhevnikov E., Kobiakov D., Guzev M., Wiercigroch M. (2021). Nonlinear Young’s Modulus of New Red Sandstone: Experimental Studies. Acta Mech. Solida Sin..

